# Genome Sequence of *Streptococcus agalactiae* Strain H002, Serotype III, Isolated in China from a Pregnant Woman

**DOI:** 10.1128/genomeA.01109-15

**Published:** 2015-10-01

**Authors:** Rui Wang, Liping Li, Fuguang Luo, Wanwen Liang, Xi Gan, Ming Chen

**Affiliations:** a Guangxi Key Laboratory for Aquatic Genetic Breeding and Healthy Aquaculture, Guangxi Academy of Fishery Sciences, Nanning, China; b Liuzhou Aquaculture Technology Extending Station, Liuzhou, China

## Abstract

Here, we report the first whole-genome sequence of *Streptococcus agalactiae* strain H002, serotype III, isolated in China from a woman 32 weeks pregnant. This sequence represents an important addition to the published genomes and will promote comparative genomic studies of *S. agalactiae* spp. isolated from diverse regions, particularly when compared with Chinese strains.

## GENOME ANNOUNCEMENT

Group B streptococcus (GBS) (*Streptococcus agalactiae*) is the most common cause of neonatal and obstetric sepsis and is an increasingly important cause of septicemia in elderly individuals and immunocompromised patients (1, 2). The serotype III GBS strain is a major pathogen responsible for the majority of infections, including meningitis, in neonates worldwide (3). In recent years, the detection rate of *S. agalactiae* in pregnant women, newborns, and elderly people is on the increase in China (4, 5). The isolate rate of *S. agalactiae* from vaginal secretions of pregnant women is between 2.7 and 11.7% (4), and it was as high as 64.7% from women patients older than 65 years in one hospital (5). Analysis of the strains serotypes and genome sequences is very important for vaccine development and drug treatment (6). The NCBI database currently has 271 published strains of the *S. agalactiae* genome assembly and annotation reports, but no one has isolated one from Chinese people. The *S. agalactiae* strain H002, serotype III, which was isolated from woman 32 weeks pregnant in Guangxi Province, China, was chosen for sequencing.

The genome sequence of *S. agalactiae* strain H002 was determined using Illumina Genome Analyzer II (GAII) at the Beijing Genomics Institute (BGI) (Shenzhen, China). The assemblies were based on 500-Mb reads. All reads provided about 98-fold coverage of the genome. The GAII paired-end reads were assembled into 54 contigs in 14 scaffolds with the SOAPdenovo program. Gaps were closed by primer walking and sequencing of PCR products. Automatic gene annotation was carried out by the NCBI Prokaryotic Genome Annotation Pipeline (released in 2013) (http://www.ncbi.nlm.nih.gov/genome/annotation_prok/) and was followed by manual editing. The complete genome of H002 is composed of a 2,147,416-bp single circular chromosome with a G+C content of 35.65%. A total of 2,013 protein-encoding genes, 30 pseudogenes, 80 tRNA genes, 21 5S-16S-23S rRNA operons, 1 noncoding RNA (ncRNA), and 14 frameshifted genes are found to be located in this chromosome. In addition, the chromosome harbors 3 prophage-like elements and 1 putative clustered regularly interspaced short palindromic repeat (CRISPR).

The H002 sequence data will be useful for future studies that aim to examine genetic variation among GBS serotype III strains from diverse regions and will provide further insight into the host adaptation and pathogenicity of this important bacterial pathogen.

### Nucleotide sequence accession number.

The genome sequence of *S. agalactiae* strain H002 reported in this paper has been deposited in the GenBank database under accession number CP011329.
